# Optical Control of High-Harmonic Generation at the Atomic Thickness

**DOI:** 10.1021/acs.nanolett.2c02711

**Published:** 2022-10-28

**Authors:** Yadong Wang, Fadil Iyikanat, Xueyin Bai, Xuerong Hu, Susobhan Das, Yunyun Dai, Yi Zhang, Luojun Du, Shisheng Li, Harri Lipsanen, F. Javier García de Abajo, Zhipei Sun

**Affiliations:** † Department of Electronics and Nanoengineering, Aalto University, Espoo02150, Finland; ‡ ICFO-Institut de Ciencies Fotoniques, The Barcelona Institute of Science and Technology, 08860Castelldefels, Barcelona, Spain; § International Cooperation Base of Photoelectric Technology and Functional Materials, and Institute of Photonics and Photon-Technology, Northwest University, Xi’an710069, China; ∥ WPI International Center for Materials Nanoarchitectonics, National Institute for Materials Science, Tsukuba305-0044, Japan; ⊥ ICREA-Institució Catalana de Recerca i Estudis Avançats, Passeig Lluís Companys 23, 08010Barcelona, Spain; # QTF Centre of Excellence, Department of Applied Physics, Aalto University, Espoo02150, Finland

**Keywords:** All-Optical Control, Static High-Harmonic Generation, Transient High-Harmonic Generation, Interband Carrier Transition, Electronic States, Real-Time Quantitative Theory of Nonlinear Optics

## Abstract

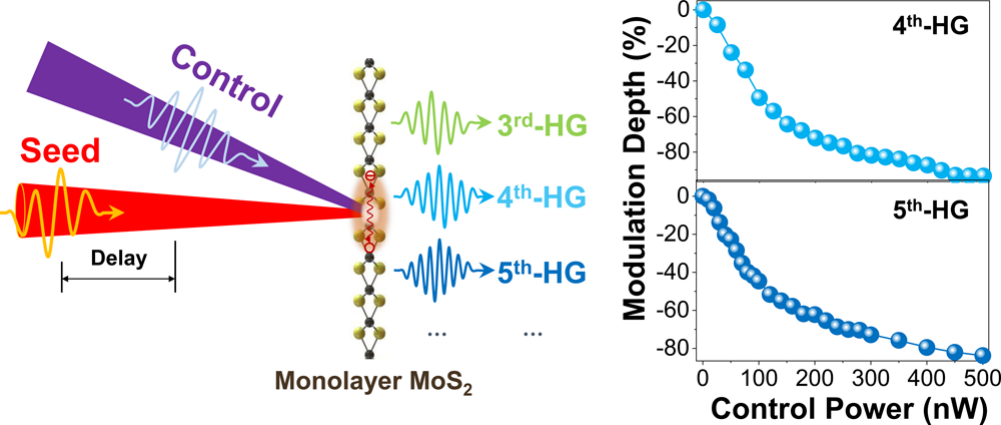

High-harmonic generation (HHG), an extreme nonlinear optical phenomenon beyond the perturbation regime, is of great significance for various potential applications, such as high-energy ultrashort pulse generation with outstanding spatiotemporal coherence. However, efficient active control of HHG is still challenging due to the weak light–matter interaction displayed by currently known materials. Here, we demonstrate optically controlled HHG in monolayer semiconductors via the engineering of interband polarization. We find that HHG can be efficiently controlled in the excitonic spectral region with modulation depths up to 95% and ultrafast response speeds of several picoseconds. Quantitative time-domain theory of the nonlinear optical susceptibilities in monolayer semiconductors further corroborates these experimental observations. Our demonstration not only offers an in-depth understanding of HHG but also provides an effective approach toward active optical devices for strong-field physics and extreme nonlinear optics.

Recently, high-harmonic generation (HHG) in solid materials^1^ has attracted significant interest in strong-field and attosecond physics. While HHG based on atomic gases has formed the basis of attosecond science, the required complex setups with vacuum operation environments and delicate optics limit different practical applications.^2^ The emerging field of solid-state HHG naturally holds a series of advantageous features, such as a compact configuration to simplify the operation conditions, the requirement of lower pulse intensities (few μJ in solids vs few mJ in gases),^3−5^ and the involvement of strong electron interactions.^4^ The prospect to open possibilities extending beyond gas-phase HHG is thus strong motivation to further develop the field of extreme solid-state photonics.^6^ Since the pioneering observation of HHG in a bulk ZnO crystal,^7^ solid-state HHG has been demonstrated in a wide range of materials, including large-bandgap dielectrics,^8,9^ semiconductors,^10−13^ semimetals,^14,15^ topological insulators,^16^ and an ε-near-zero material.^17^ These results have revealed that HHG not only provides an effective method to probe the electronic structure in solids within the regime of strong interaction with light fields^18−20^ but also constitutes a promising platform for novel spectroscopy and microscopy techniques in advanced photonics and optoelectronics.^4,21^

Active control over the extreme nonlinear processes involved in HHG would be a concrete step toward various applications. Currently, solid-state HHG is commonly understood as the result of two prominent physical mechanisms: interband polarization and light-driven intraband currents.^5,22−25^ Both mechanisms offer plausible opportunities to control HHG by manipulating the interband polarization or intraband current. For example, the HHG efficiency in bulk ZnO has been tuned by incoherently driving photoexcited carriers.^26^ Electrically controlled HHG in carbon nanotubes has also been reported by tuning the electron and hole doping levels.^27^ In addition, solid-state HHG has been engineered with symmetric^21^ or artificial structures,^17,28,29^ such as metallic plasmons and dielectric nanostructures. These abilities to tune or enhance HHG emphasize the potential of actively controlling HHG. The superior features of all-optical control (e.g., ease of operation, high speed, and low-power consumption) are expected to facilitate ultrafast optoelectronic applications,^30−36^ such as optical coding and data processing.^37,38^ Moreover, measurements of HHG in the time domain could play a fundamental role in understanding the physical mechanisms underlying the extreme optics of solid-state materials.^6,39^ However, thus far, efficient control of HHG in solids remains elusive.

Recently, two-dimensional layered materials (2DLMs), which hold the advantages of strong optical nonlinear response and automatic fulfillment of the phase matching condition, have been found to serve as a powerful platform for HHG without the need to account for propagation effects, and thus, they are appealing for extreme nonlinear optics.^12,14,40−43^ Because of the strong interband oscillation strength in these materials, HHG in 2DLMs can be dramatically and resonantly enhanced by electronic states.^11^ All-optical active control of HHG from 2DLMs thus appears as an exciting opportunity.

In this work, we demonstrate an efficient optical control of HHG in monolayer MoS_2_ and few-layer graphene. All-optical control is realized by tuning the interband polarization strength via the valence carrier density, achieving a high modulation depth of ∼95% in monolayer MoS_2_ at an incident pulse energy of 250 pJ and an ultrafast response time within the subpicosecond regime in few-layer graphene. Further, we present a real-time quantitative theory of nonlinear optical susceptibilities illustrating the underlying carrier dynamics. Our experimental and theoretical results show that interband polarization constitutes an effective method to modulate HHG in 2DLMs.

The monolayer MoS_2_ flake used in our experiment is grown by chemical vapor deposition (CVD), and its quality is confirmed by photoluminescence and Raman spectroscopies (see Figure S1 in the Supporting Information). The nonlinear optical response in monolayer MoS_2_ is analyzed at room temperature when illuminated with femtosecond seed pulses (∼150 fs, 2 kHz, photon energy tunable in a range of ∼0.52 to 0.78 eV). To modulate the nonlinear optical response of the seed light in monolayer MoS_2_, we employ the control light with a photon energy ℏω_c_ of ∼3.1 eV, as shown in Figure 1a. Figure 1b shows the HHG spectra obtained with and without the control light when the incident seed photon energy ℏω_0_ is ∼0.57 eV with an average power of ∼80 μW and a delay time Δτ ≈ 0.4 ps. In the HHG spectrum, the third-, fourth-, and fifth-harmonic generations (HGs) are observed at the photon energies of ∼1.71, 2.29, and 2.85 eV, respectively. With the control light (incident power of ∼100 nW), the fourth- and fifth-harmonic signals significantly decrease, while the third-harmonic signal remains constant (see time-resolved 3^rd^-HG in Figure S2). This indicates that the harmonic signal is not affected by the interband carrier density when the photon energy is below the optical bandgap of monolayer MoS_2_.^34,35^ To discuss the optical modulation of HHG in more detail, we focus on the fourth- and fifth-harmonic signals below.

**Figure 1:**
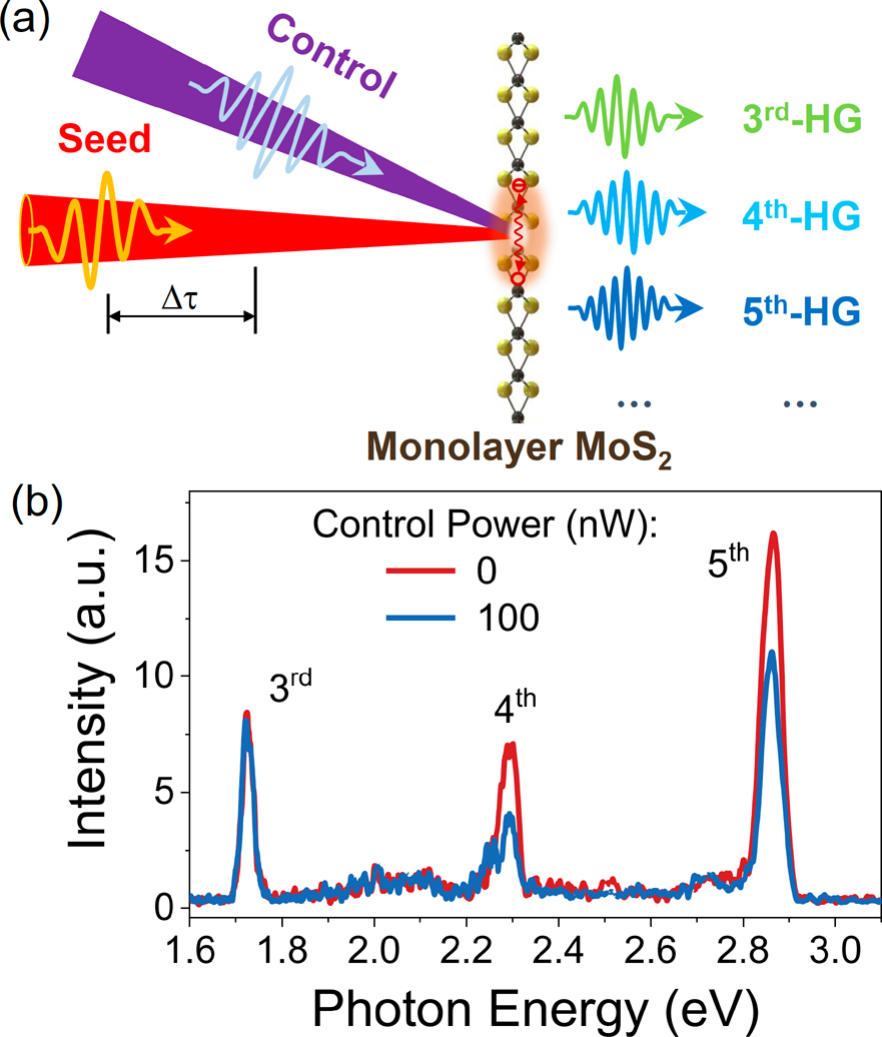
All-optical control of HHG in a 2DLM. (a) Schematic illustration of optically controlled HHG in monolayer MoS_2_. HHG produced by excitation of the seed light in monolayer MoS_2_ is modulated via exposure to the control light. (b) HHG spectra revealing the intensity associated with third, fourth, and fifth harmonics in the presence (blue) and absence (red) of the control light. The photon energy of control light is ∼3.1 eV with an average power of ∼100 nW (corresponding peak intensity *I*_c_ ∼ 1.44 GW/cm^2^), and the seed light photon energy is ∼0.57 eV with an average power of ∼80 μW (corresponding peak intensity *I*_0_ ∼ 1.15 TW/cm^2^). The time delay Δτ between the seed and control light pulses is ∼0.4 ps.

First, we study the static fourth-order HHG (i.e., the control light is off). Figure 2a shows the fourth-harmonic spectrum when the seed photon energy ℏω_0_ is set to ∼0.71 eV (wavelength λ_0_ = 1760 nm) with an average power (*P*_0_) of 45 μW. The output photon energy is centered at ℏω_4th_ ≈ 2.84 eV (*λ*_4th_ ≈ 437 nm), confirming the fourth-harmonic generation process. Remarkably, the fourth-harmonic output power follows a power law *P*_0_^α^ with an index α of 3 when the average seed light power is *P*_0_ ≤ 60 μW (Figure 2b). This power dependence shows a lower slope than the power law of *P*^4^ that is typical of the perturbative regime (i.e., with a fourth-harmonic nonlinear polarization *p*^(4ω)^ ≈ ε_0_χ^(4)^|**E**(ω_0_)|^4^, where ε_0_ is the permittivity of free space, χ^(4)^ corresponds to the fourth-order nonlinear optical susceptibility, and **E**(ω_0_) denotes the electric field of the incident seed-light beam, respectively^44^). Figure 2c shows the peak intensities of the static 4^th^-HG spectra via tuning the seed photon energy from 0.60 to 0.74 eV with *P*_0_ fixed at 45 μW. The results show a power variation (*P*_max_/*P*_min_) around 16 with a prominent peak at ∼2.85 eV and a small peak at ∼2.43 eV. We assign the peak at 2.85 eV to the C exciton formed by the band-nesting effect in monolayer MoS_2_.^11,45^ The peak at 2.43 eV could be caused by the other resonantly excited exciton states (such as the 3*s* exciton^46^).

**Figure 2:**
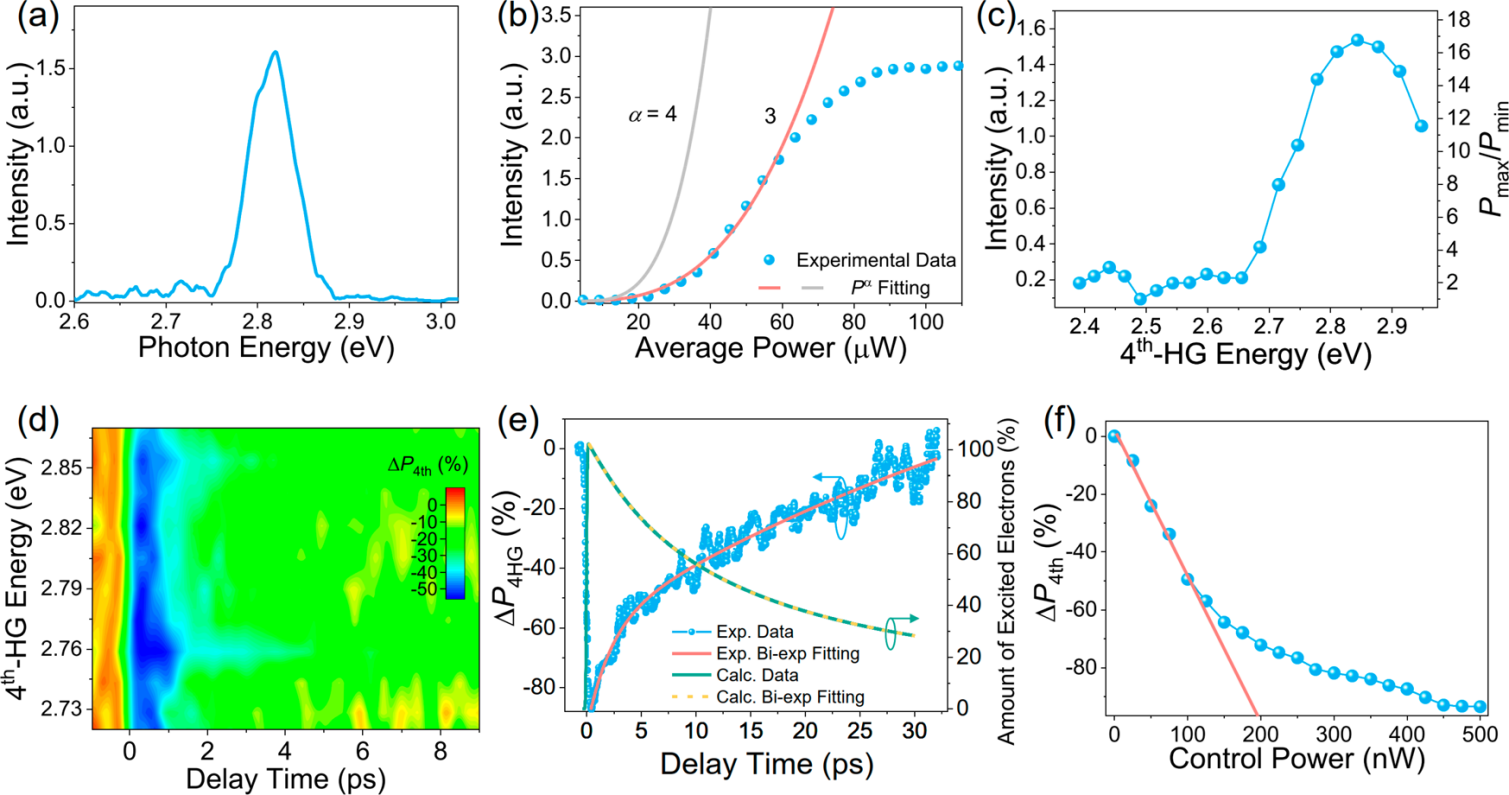
All-optical control of 4^th^-HG in monolayer MoS_**2**_. (a) Light emission intensity spectrum around the 4^th^-HG spectrum when the seed photon energy is ∼0.71 eV. (b) Dependence on seed power *P*_0_ of the static 4^th^-HG signal (i.e., without control light) when ℏω_0_ = 0.71 eV, along with fits to *P_0_*^α^ for α = 3 (red) and α = 4 (gray). (c) Broadband static 4^th^-HG peak intensity. (d) Transient 4^th^-HG signal with the generated photon energy ranging from ∼2.72 to 2.87 eV when the control light power is *P*_c_ = ∼ 100 nW (*I*_c_ ∼ 1.44 GW/cm^2^). (e) Transient 4^th^-HG signal (blue curve) along with a biexponential fit (red curve) when ℏω_4th_ ≈ 2.85 eV and *P*_c_ ≈ 400 nW. The theoretically calculated temporal variation of the number of excited electrons in the conduction band is included for comparison (green curve), which is well fitted by a biexponential function (yellow dashed curve). (f) Power dependence of the transient 4^th^-HG signal when Δτ ≈ 0.4 ps, where the red line is a linear fit at low control power. We fix the seed light power to *P*_0_ ≈ 45 μW (*I*_0_ ∼ 0.65 TW/cm^2^) during experiments in (a) and (c)–(f).

To achieve optically controlled 4^th^-HG, we now introduce the control light with a photon energy (ℏω_c_) of 3.1 eV and an average power (*P*_c_) of 100 nW. Figure 2d shows the transient fourth-harmonic signal (Δ*P*_4th_) generated from 2.7 to 2.87 eV versus the delay time between the control and seed pulses. We apply the definition Δ*P*_4th_ = (*P*_w_ – *P*_wo_)/*P*_wo_, where *P*_w_ and *P*_wo_ denote the fourth-harmonic signal power with and without the control light, respectively. Note that the controlled 4^th^-HG at other wavelength regions is beyond our detection ability due to the relatively weak fourth-harmonic signal (Figure 2c). Nevertheless, the results demonstrate a broadband control of 4^th^-HG around the band-nesting region of monolayer MoS_2_. Figure 2e shows Δ*P*_4th_ at the 4^th^-HG energy of 2.85 eV as a function of delay time (Δτ) between the control and seed pulses. We find that Δ*P*_4th_ first decreases within 100 fs due to optical bleaching and subsequently starts to recover at ∼30 ps. The biexponential fitting of the recovering trace yields fast and slow time constants of τ_1_ ≈ 2.2 ± 0.16 ps and τ_2_ ≈ 57.6 ± 10 ps, respectively. We note that the carrier dynamics are independent of the control light power. Further, we examine the control power dependence of Δ*P*_4th_ when Δτ ≈ 0.4 ps (Figure 2f), from which we find that Δ*P*_4th_ decreases linearly and sharply to a value of −65% with a control power of ∼150 nW and then slowly drops to −95% when the control power is increased to ∼500 nW (corresponding to a pulse energy of 250 pJ). This indicates full control over the 4^th^-HG intensity with the control light. The linear modulation dependence on the control light power can be attributed to the fact that the control light linearly excites the electrons in the valence band when its power is small.

Now, we focus on the 5^th^-HG in monolayer MoS_2_. Figure 3a shows the static 5^th^-HG spectrum of monolayer MoS_2_ when the seed photon energy (ℏω_0_) is 0.565 eV with an average power (*P*_0_) of 50 μW. The peak output photon energy of the 5^th^-HG is at 2.80 eV, confirming its 5^th^-HG nature. When increasing the seed light power, the intensity of the 5^th^-HG signal increases nonlinearly (Figure 3b). It follows a power index of α ≈ 3.83 when the incident optical power is ≤80 μW, then starts being saturated. Similar to the 4^th^-HG, we find that the 5^th^-HG signal shows a large deviation in power dependence from the *P_0_*^5^ behavior expected in the perturbative regime (i.e., with a fifth-harmonic nonlinear polarization *p*^(5ω)^ ≈ ε_0_χ^(5)^|**E**(ω_0_)|^5^, where the χ^(5)^ corresponds to the fifth-order nonlinear optical susceptibility). To investigate such deviation, we measure the wavelength-dependence of the 5^th^-HG signal. As shown in Figure 3c, the *P*_max_/*P*_min_ ratio is as high as ∼83 with several strong resonance peaks in the range of 2.46–3.30 eV. The highest peak is at ∼2.85 eV, close to the C exciton state formed by the band-nesting effect, indicating a strong enhancement of 5^th^-HG assisted by coupling to the C exciton. The peak at ∼3.04 eV is not prominent, so we do not discuss it here.^47^ Another strong peak is observed at ∼2.6 eV and could arise from other excited exciton states or the electronic band edge, although its origin is still unclear.

**Figure 3:**
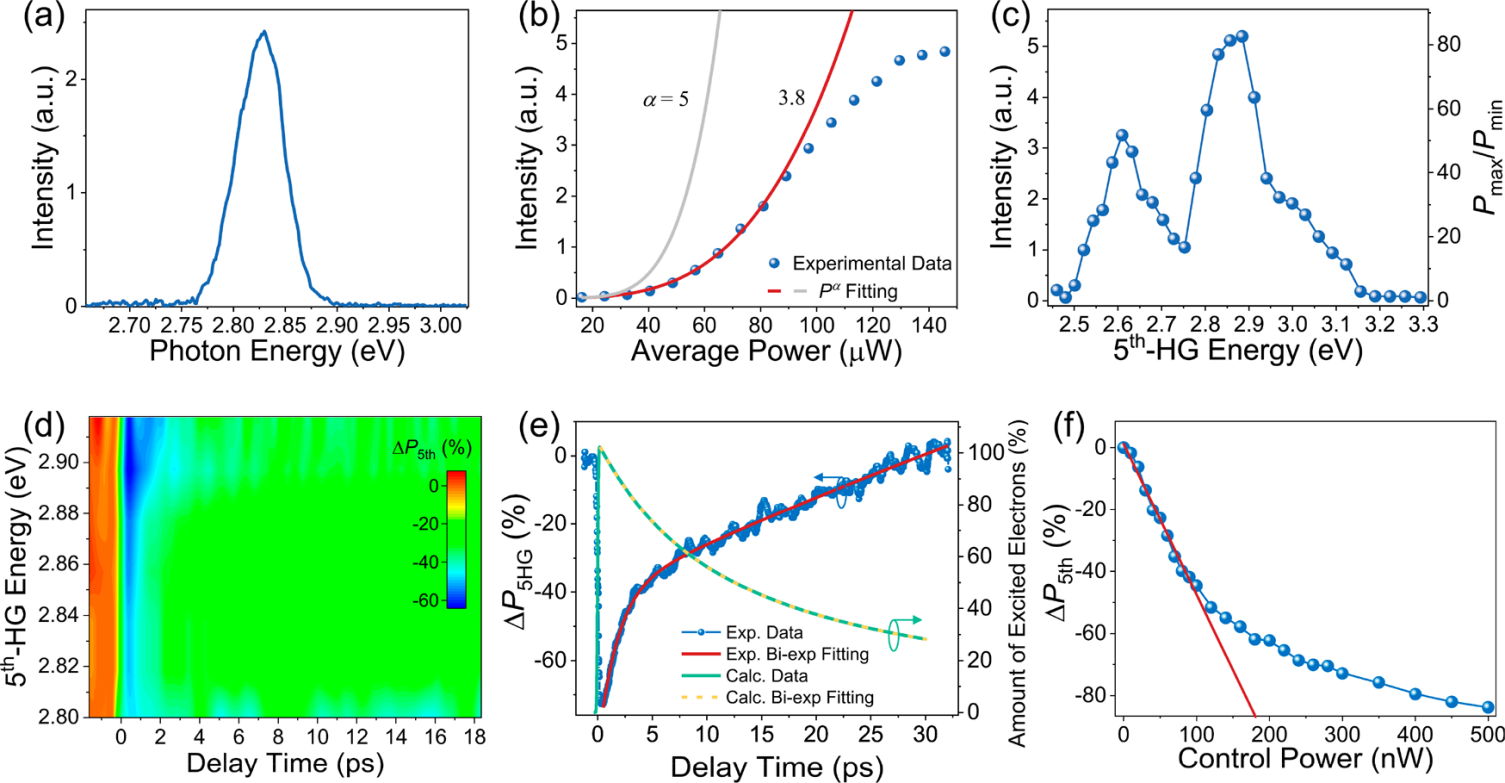
All-optical control of 5^th^-HG in monolayer MoS_2_. (a) Static 5^th^-HG spectrum when the seed laser photon energy is ℏω_0_ = 0.565 eV. (b) Power dependence of static 5^th^-HG signal with ℏω_0_ = 0.565 eV, where the solid lines are fitting curves with *P_0_*^α^, for α = 3.8 (red) and α = 5 (gray). (c) Broadband static 5^th^-HG signal peak intensity as a function of output photon energy. (d) Transient 5^th^-HG signal with generated photon energies ranging from 2.8 to 2.92 eV. (e) Transient 5^th^-HG signal when ℏω_5th_ = 2.85 eV and *P*_c_ = 300 nW, which is fitted by a biexponential function (red curve). The theoretically calculated temporal variation of the number of excited electrons in the conduction band is included for comparison (green curve), fitted by a biexponential function (yellow dashed curve). (f) Power dependence of the optically controlled 5^th^-HG signal, where the red line is a linear fit at low control power. During the experiments, the seed light power is fixed at *P*_0_ = 50 μW (*I*_0_ ∼ 0.72 GW/cm^2^) in (a) and (c)–(f).

Further, we carry out experiments to investigate optically controlled 5^th^-HG with the control light at 3.1 eV. Figure 3d shows the time-resolved broadband modulation of the 5^th^-HG signal (Δ*P*_5th_) from ∼2.80 to 2.92 eV with an average control power *P*_c_ ≈ 100 nW. The 5^th^-HG signal can be strongly bleached with the control light, and the highest modulation depth has been found at a generated photon energy of ∼2.90 eV, close to the C exciton. To observe the dynamics, we extract the time-resolved modulation of the 5^th^-HG at a generation photon energy of ∼2.85 eV. As shown in Figure 3e, Δ*P*_5th_ behaves similarly to Δ*P*_4th_, first decreasing sharply within 100 fs and then recovering with two different time constants (τ_1_ ≈ 1.7 ± 0.04 ps and τ_2_ ≈ 143.2 ± 26 ps). Figure 3f indicates that the fifth-harmonic modulation Δ*P*_5th_ first decreases linearly when the control power is below ∼100 nW, and then tends to be saturated when increasing the control power. The maximum modulation depth can reach up to −84% with a control light power of ∼500 nW (*I*_c_ ∼ 7.18 GW/cm^2^).

The underlying mechanism of solid-state HHG is generally described as a combined effect of interband polarization and intraband oscillations.^4^ Especially, when the generated harmonic photon energy is larger than the bandgap, the solid-state HHG operating with significant overlap of atomic orbitals gives rise to a probability of interband polarization, which we attribute as the mechanism explaining our experimental results: HHG is highly dependent on the amount of valence carrier states, and thus its strength is modified by interband excitations. This is illustrated by our HHG measurements (Figures 2c and 3c), which support a large enhancement at the C exciton. The observable enhancements of 4^th^-HG and 5^th^-HG in amplitude are as high as ∼17 and ∼83 times compared to the lowest measured nonlinear signal wavelengths. The deviation in the power dependence from the perturbative power-law *P*_0_^α^ with exponents α of 4 and 5 for 4^th^-HG- and 5^th^-HG (see Figures 2b and 3b) further points to a dominant effect played by multiphoton state-related resonances. With increasing seed-light power, the HHG signal tends to be strongly saturated (Figures 2b and 3b): a clear multiphoton saturable absorption effect is observed.^48^

We understand that by illuminating with control light, a redistribution of valence carriers is produced, which affects HHG and, thus, enables its modulation. More precisely, we attribute this effect to the bleaching of valence carriers by the control light (i.e., promotion to the conduction band), which reduces the interband polarization strength and, therefore, decreases the HHG efficiency. This mechanism supported by our time-resolved HHG measurements in Figures 2e and 3e clearly shows that the bleaching process happens very fast within 100 fs. Subsequently, HHG starts recovering with two different time constants along with the relaxation of photocarriers. The fast time constant can be understood as the result of carrier relaxation processes, while the slow time constant should be associated with slower electron–hole recombination processes.^34,49,50^ Our results for the dependence of the modulation on the power of the control light (Figures 2f and 3f) also confirm that the bleaching of valence carriers with the control light can significantly reduce the HHG intensity. Considering the high modulation depth of 4^th^-HG (5^th^-HG) by as much as 95% (84%), we further corroborate that interband polarization contributes dominantly to HHG in the explored energy region. In our experiment, we also extend our optical control method to HHG in graphene (see Figures S5 and S6 in section 8 of Supporting Information). Compared to the transient responses in MoS_2_, the decay time of photon carriers in graphene is much faster because of its higher carrier relaxation rate, while the modulation depth in graphene is relatively small due to its low carrier density. This further highlights the importance of the coupling to the exciton states for large modulation in MoS_2_.

To fully understand these experimental results, we calculate the high-order nonlinear optical response of monolayer MoS_2_. In the past, the intrinsic complexity of the nonlinear optical response of nanomaterials has stimulated the development of quantitative theoretical prediction capabilities. Indeed, advanced theoretical approaches have been introduced in recent years to simulate nonlinear optical phenomena, such as second-harmonic generation (SHG)^51,52^ and third-harmonic generation (THG),^31,35^ and formulate predictions in good agreement with experimental observations. Here, we use density functional theory in a real-time approach to calculate *ab initio* the nonlinear optical response of monolayer MoS_2_. In particular, we use many-body perturbation theory to accurately obtain the electronic structure of the material within the G_0_W_0_ approximation^53,54^ and further include excitonic effects by solving the Bethe–Salpeter (BS) equation^55,56^ as implemented in the YAMBO code.^57^ Subsequently, we perform real-time simulations to predict the spectral dependence of the nonlinear susceptibilities associated with harmonic generation at second-to-fifth orders.

To understand the nonlinear optical response of the material in detail, we first discuss optical transitions captured by the linear dielectric function. The electronic structure and absorption spectrum of the material (see Supporting Information for parameter sets) are shown in Figure S3. The G_0_W_0_ method produces a direct electronic bandgap of ∼2.71 eV in monolayer MoS_2_. In addition, BS calculations for the spectral positions of the optical band-edge excitons 1*s*_A_ and 1*s*_B_ yield 2.04 and 2.18 eV, respectively, while the 1*s*_C_ exciton is also located below the bandgap at ∼2.69 eV, and contributions from higher-order excitonic states ∼2.41 eV are discernible in the spectrum.

Figure 4a,b shows our simulated spectra for second-to-fifth harmonic generation from monolayer MoS_2_ over a broad spectral frequency range. It is worth noting that the reduced spatial symmetry of the monolayer enables the generation of even-order harmonics in addition to odd-order ones, in contrast to bulk and even-layered systems of the same crystal.^58^ In particular, the SHG spectrum (blue curve in Figure 4a) is in excellent agreement with previous experimental^59^ and theoretical^60^ results, thus demonstrating the reliability of our calculation methods. Second- and higher-order harmonics in monolayer MoS_2_ exhibit multiple resonances associated with excitonic states, whose spectral positions are visible in the absorption spectrum (Figure 4a,b). More precisely, we find that the highest third- and fifth-harmonic signals (|χ^(3)^| ≃ 3.8 × 10^–18^ m^2^/V^2^ and |χ^(5)^| ≃ 6.6 × 10^–36^ m^4^/V^4^, respectively) originate in coupling to A and B excitons, while the strongest even-order harmonic responses are stemming from the 1*s*_C_ exciton (|χ^(2)^| ≃ 4.8 × 10^–9^ m/V and |χ^(4)^| ≃ 1.1 × 10^–27^ m^3^/V^3^). These results agree with previous observations of high even-order harmonic response in the band nesting regions of monolayer TMDs.^11^ To more clearly illustrate this, we calculate the relative output intensities for harmonic generation in resonance with 1*s*_A_, 1*s*_B_, and 1*s*_C_ excitons under the same excitation intensity (see Supporting Information for details), as shown in Figure 4c. While all excitons show comparable output intensities for odd-ordered harmonics, 1*s*_C_ excitons are found to be more efficient at even orders. In addition, we also study the output intensities observed at resonance with the 1*s*_C_ exciton (our experimental setup allows us to obtain systematic results only around the 1*s*_C_-exciton region). While theoretical and experimental output intensities are comparable for SHG, the results differ more as the harmonic order increases, although the qualitative trend is similar in both theory and experiment. Note that different excitation intensities would induce variations in efficiency due to the different order power dependence of the nonlinear optical signals.

**Figure 4:**
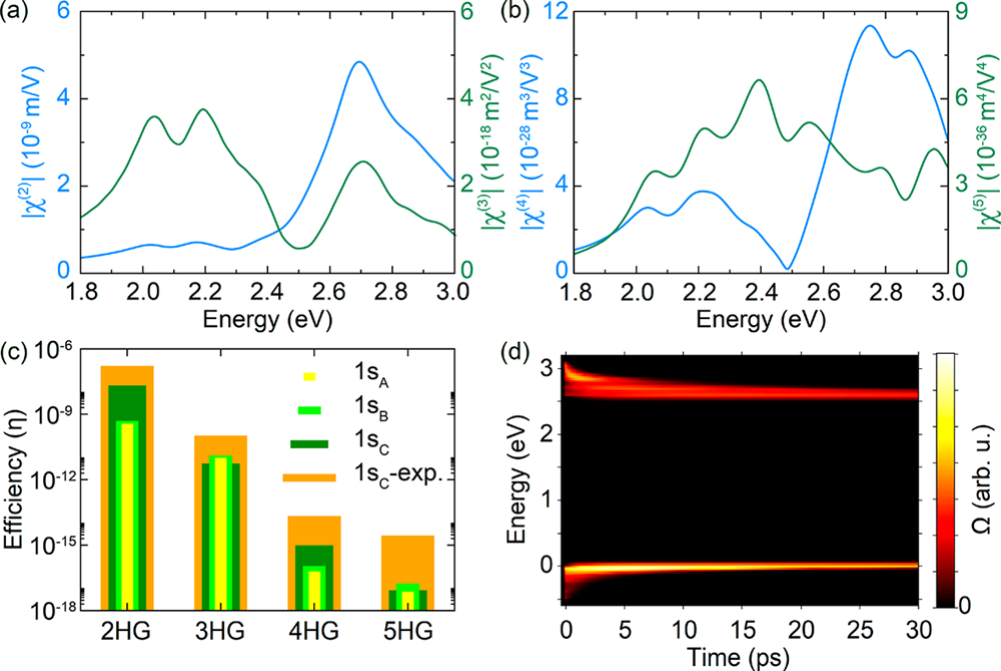
High-order nonlinear optical responses in monolayer MoS_2_. (a, b) Spectral dependence of the nonlinear susceptibilities of monolayer MoS_2_ corresponding to second-to-fifth harmonic generation (see vertical axis labels), as calculated within the Bethe–Salpeter-equation method on top of the G_0_W_0_ electronic structure. (c) Calculated relative intensities associated with 1*s*_A_, 1*s*_B_, and 1*s*_C_ excitons for second-to-fifth harmonics (see color-matched legend), compared with the experimental relative intensities of harmonics at the 1*s*_C_ exciton (orange). (d) Variation of the electron (in conduction bands) and hole (in valence bands) occupations (Ω) as a function of time and energy using the rate equation model.

To obtain a microscopic understanding of the time-dependent HHG process, we further calculate the electronic-level occupation dynamics in the system based on first-principles parameters and phenomenological decay rates. In particular, we simulate the transition process of charge carriers excited by the control laser field to the ground level by radiative and nonradiative recombination events. The number and distribution of excited electrons in the conduction bands evolve in time according to the rate equation
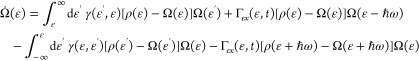
where ρ(ε) and Ω(ε) denote the density of states (see Figure S4a) and the electron occupation density as a function of electron energy ε, respectively. Also, Γ_*ex*_(ε,t) = (2*πe*^2^*E*_0_^2^/ℏ)e^–2*t*^2^/Δ^2^^*d*_ε,ε–ℏω_^2^ describes the excitation rate associated with the electric-field amplitude of the external control light *E*_0_. We consider excitation by a laser pulse with Δ = 150 fs duration centered around a photon energy ℏω = 3.1 eV. In Γ_*ex*_(ε,*t*), we introduce *d*_ε,ε–ℏω_^2^ as the Brillouin zone (BZ) average of the squared transition dipole matrix elements between states with energies ε and ε – ℏω. We consider two types of processes contributing to the recombination rate, radiative and nonradiative, so we have γ = γ_*rad*_ + γ_nonrad_. The nonradiative decay rate γ_nonrad_ is considered to be 1/(100 fs) for transitions with energies less than 0.2 eV as a phenomenological way of incorporating intraband processes assisted by phonon creation. In addition, for the radiative decay rate, we consider  which describes recombination assisted by the emission of one photon.^61^ Unfortunately, this expression for γ_rad_(ε,ε′) results in recombination times that are much longer than those observed experimentally (∼30 ps), thus indicating that additional recombination mechanisms other than photon emission must be dominant and in particular recombination assisted by exciton creation.^62,63^ In this line, Auger electron–hole recombination assisted by bound excitons has been observed in MoS_2_,^64,65^ while defect-assisted recombination is known to affect the recombination rates in this material as well.^66^ Assuming a similar role of the transition matrix elements as in radiative recombination, we can phenomenologically account for exciton-mediated recombination by correcting γ_*rad*_(ε,ε′) through a multiplicative factor 3 × 10^3^, which we determine by comparison with the experimental results. This argument is supported by the high local density of optical states at the layer position, dominated by coupling to excitons, as argued in section 7 of the Supporting Information. The resulting temporal evolution of the charge carrier energy distributions is plotted in Figure 4d, where we observe that shortly after the control laser is applied, the excited holes are grouped within a small energy window at the top of the valence band via fast transitions. In addition, the electron occupation of the conduction bands obtained from this model (dashed-yellow curves in Figures 2e and 3e) are in good correspondence with the experimental observations and the biexponential fit.

In conclusion, we have demonstrated active control of HHG at various orders in materials of atomic thickness. A strong enhancement of HHG (∼83 times) has been determined for monolayer MoS_2_ at harmonics resonating with the 1*s*_C_ excitonic state. By optically modifying the distribution of charge carriers, HHG in monolayer MoS_2_ and few-layered graphene has been controlled with high modulation depth (up to 95%), low power consumption (250 pJ), ultrafast speed (several picoseconds), and small footprints (atomic thickness). Further, we have developed a quantitative theoretical analysis for the nonlinear optical responses associated with second-to-fifth-order processes, as well as the HHG efficiency and its relation to the temporal evolution of the carrier density distribution. Our demonstration of optically controlled HHG paves a new route for engineering extreme nonlinear optics in atomically thin materials with potential application in active optical devices.

## Supporting Information Available

(1) Optical properties of monolayer MoS_2_; (2) time-resolved THG modulation below the bandgap; (3) computational details; (4) derivation of relative intensities; (5) carrier dynamics; (6) calculation of density of states and dipole matrix elements; (7) enhancement of recombination transition rates by coupling to excitons; (8) optically controlled HHG in few-layer graphene (PDF)
