# Supplementary material for: Optical Control of High-Harmonic Generation at the Atomic Thickness
Source: Nano Lett. 2022 Oct 28;22(21):8455–62. doi: 10.1021/acs.nanolett.2c02711 (PMC9650768; doi:10.1021/acs.nanolett.2c02711)
Supplement: Supplementary file 1 — nl2c02711_si_001.pdf [file nl2c02711_si_001.pdf]

Supplementary Information for

"Optical control of high-harmonic generation at the atomic thickness"

Yadong Wang, Fadil Iyikanat, Xueyin Bai, Xuerong Hu, Susobhan Das, Yunyun Dai, Yi Zhang, Luojun Du, Shisheng Li, Harri Lipsanen, F. Javier García de Abajo*, and Zhipei Sun**

*E-mail: zhipei.sun@aalto.fi, javier.garciadeabajo@nanophotonics.es, yadong.wang@aalto.fi

1. Optical properties of monolayer MoS_2

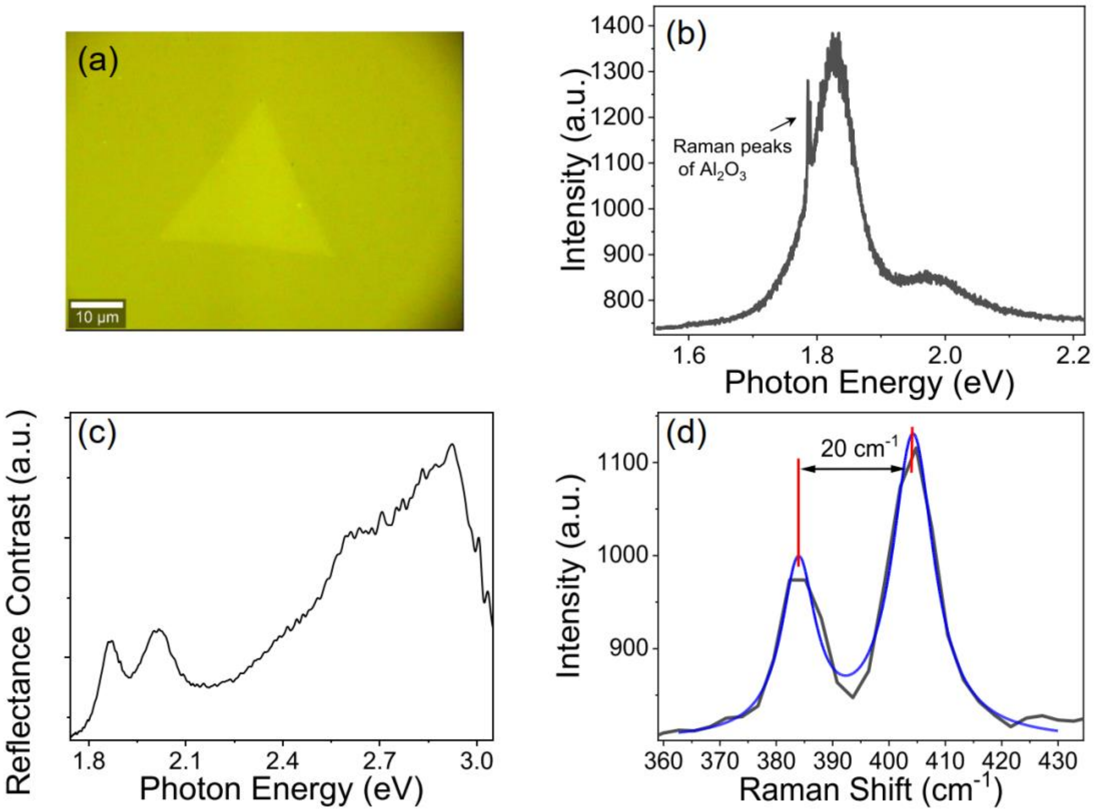

Figure S1. (a) Optical image of CVD-grown monolayer MoS_2. (b) Photoluminescence of monolayer MoS_2. (c) Reflectance contrast of monolayer MoS_2. (d) Raman spectrum of monolayer MoS_2, which is fitted by a multi-Lorentzian function (blue curve). The difference between the two peaks is around 20 cm^{-1}, indicating a monolayer thickness.

2. Time-resolved THG modulation lower than the bandgap

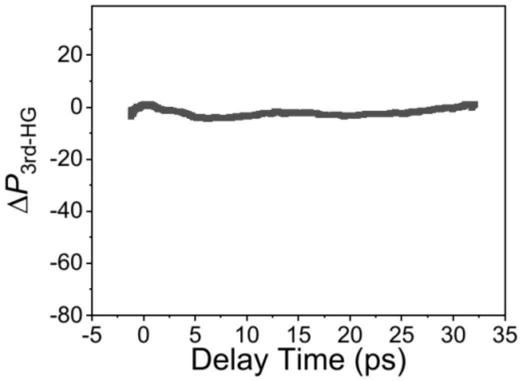

Figure S2. Time-resolved 3rd-HG signal when $\hbar\omega_0 \approx 0.57$ eV and $P_c = 300$ nW.

3. Computational details

We obtain the ground-state Kohn-Sham electronic structure and eigenvalues using the Quantum Espresso package [1], based on density functional theory. We use fully relativistic, norm-conserving Vanderbilt pseudopotentials [2, 3] with the Perdew-Burke-Ernzerhof (PBE) approximation [4] for the electron exchange and correlation. The Brillouin Zone (BZ) is sampled with 18×18×1 grid points. To accurately predict the optical spectrum, we include spin-orbit interaction during all parts of the calculations. Plane waves with a cutoff of 80 Ry are used, combined with a 36 a.u. supercell in the out-of-plane direction to simulate an isolated MoS_2 monolayer.

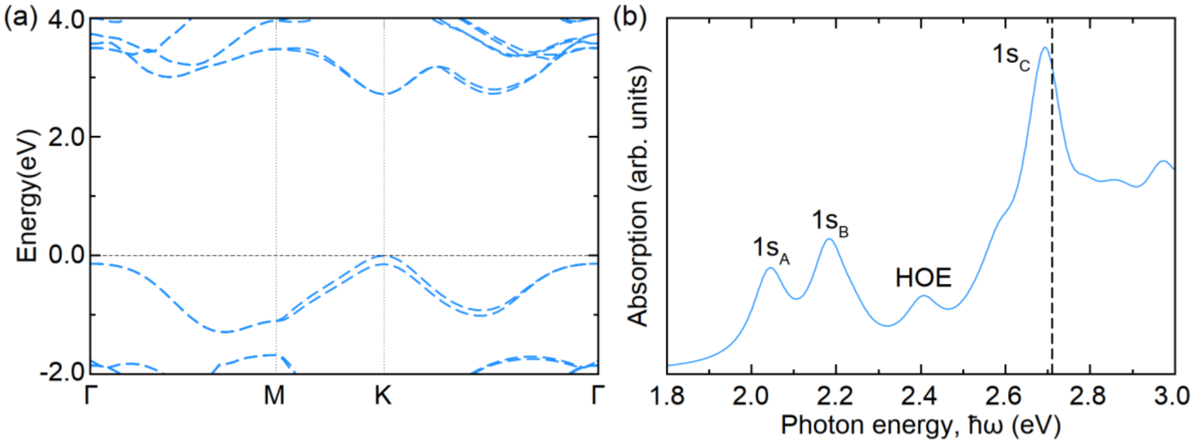

Figure S3. (a) Electronic band structure of monolayer MoS_2 within the G_0W_0 approach. (b) Optical absorption spectrum calculated for this material within the Bethe-Salpeter-equation method.

Next, the Kohn-Sham eigenenergies are corrected by the quasiparticle self-energy contribution using many-body perturbation theory within the G_0W_0 scheme, combined with the plasmon-pole approximation [5, 6] as implemented in YAMBO package [7, 8]. A relatively large **k**-point grid of size 30×30×1 is used to calculate the quasiparticle band structure. Excitonic effects are

incorporated in our calculations by solving the Bethe-Salpeter equation (BSE) [9, 10] on top of the G_0W_0-corrected eigenenergies according to the expression

$$(\varepsilon_{\mathrm{m}\mathbf{k}} - \varepsilon_{\mathrm{n}\mathbf{k}})A^{\mathrm{s}}_{\mathrm{mn}\mathbf{k}} + \sum_{\mathrm{m'n'}\mathbf{k}'} \langle \mathrm{mn}\mathbf{k}|K_{\mathrm{eh}}|\mathrm{m'n'}\mathbf{k}'\rangle A^{\mathrm{s}}_{\mathrm{m'n'}\mathbf{k}'} = \Omega^{\mathrm{s}} A^{\mathrm{s}}_{\mathrm{mn}\mathbf{k}},$$

where m and n are valence and conduction band indices, respectively; $\varepsilon_{\mathrm{m}\mathbf{k}}$ and $\varepsilon_{\mathrm{n}\mathbf{k}}$ denote the valence and conduction band quasiparticle energies, as obtained within the G_0W_0 approximation; $A^{\mathrm{s}}_{\mathrm{mnk}}$ and $\mathbf{\Omega}^{\mathrm{s}}$ are the coefficients and energies of the excitonic states, respectively; and K_{eh} is the electron-hole interaction kernel. The self-energy and dynamical dielectric screening are calculated with 150 bands. The four highest valence bands and four lowest conduction bands are included in the calculation of excitonic states. We use a customary cutoff technique to truncate the Coulomb interaction at the edges of the unit cell along the out-of-plane direction and so eliminate spurious interactions between adjacent supercells [11]. We plot the electronic band structure calculated within the G_0W_0 approach in Fig. S3a, while the absorption spectrum obtained from the BSE method is shown in Fig. S3b.

To calculate the nonlinear susceptibilities, we solve the time-dependent Schrödinger equation by carrying out real-time simulations in which the coupling between electrons and the external field, $\mathbf{E}^{\mathrm{ext}}$, is described by means of the Berry-phase formulation of dynamical polarization [12, 13]. To obtain the time dependence of the occupied valence Bloch states $|\mathrm{v}_{\mathrm{m}\mathbf{k}}\rangle$, we integrate the equation of motion

$$i\hbar\frac{\partial}{\partial t}|\mathrm{v}_{\mathrm{m}\mathbf{k}}\rangle = \left(\hat{\mathcal{H}}^{\mathrm{sys}}_{\mathbf{k}} + ie\mathbf{E}^{\mathrm{ext}}\cdot\nabla_{\mathbf{k}}\right)|\mathrm{v}_{\mathrm{m}\mathbf{k}}\rangle,$$

where the $\mathbf{E}^{\mathrm{ext}}\cdot\mathbf{\nabla}_{\mathbf{k}}$ term accounts for electron coupling to the external electric field, whereas $\hat{\mathcal{H}}^{\mathrm{sys}}_{\mathbf{k}}$ is the Hamiltonian of the unperturbed material, including the previously calculated G_0W_0 quasiparticle energies and BSE states resulting from the electron-hole interaction. Using the YAMBO package, we solve the equation of motion by employing the Crank-Nicolson method [14] with a 0.01 fs time step for a total simulation time of 100 fs for second- and third-harmonic generation, and 260 fs for fourth and fifth harmonics. We employ a phenomenological damping of 0.07 eV. Subsequently, using the obtained valence states, the time-dependent polarization of the system is obtained using the formula

$$P_{\parallel} = -\frac{e|\mathbf{a}|}{2\pi\Omega N_{\mathbf{k}_\perp}}\sum_{\mathbf{k}_\perp} \mathrm{Im}\left\{\log\left[\prod_{i=1}^{N_{k_\parallel}-1} \det S(\mathbf{k},\mathbf{k}+\mathbf{q}_\parallel)\right]\right\}$$

for the polarization of the system $P_{\parallel}$ along the lattice vector $\mathbf{a}$. Here, Ω is the volume of the unit cell; $N_{\mathbf{k}_\parallel}$ and $N_{\mathbf{k}_\perp}$ are the number of $\mathbf{k}$ points in the plane along and perpendicular to the polarization direction; we introduce the wave vector $\mathbf{q}_\parallel = \mathbf{b}/N_{k_\parallel}$ with the reciprocal lattice vector $\mathbf{b}$ chosen such that $\mathbf{b}\cdot\mathbf{a} = 2\pi$; and $S(\mathbf{k}, \mathbf{k} + \mathbf{q}_\parallel)$ is the overlap matrix between the time-dependent states $|v_{m\mathbf{k}}\rangle$ and $|v_{m\mathbf{k}+\mathbf{q}_\parallel}\rangle$. The nonlinear susceptibilities are extracted from the power series of the macroscopic polarization $\mathbf{P} = \chi^{(1)}\mathbf{E} + \chi^{(2)}\mathbf{E}^2 + \chi^{(3)}\mathbf{E}^3 + \cdots$. We consider a laser field polarized along the y direction with an intensity of 10^9 W/cm^2. The nonlinear response is computed along the same direction. To obtain vacuum-independent results, we rescale the calculated nonlinear susceptibilities by the ratio of the bulk effective thickness to the length of the out-of-plane lattice vector.

4. Derivation of relative intensities

To obtain the relative amplitudes of the fields associated with harmonic generation under normal incidence, $E_n(z) = A_n \mathrm{e}^{\mathrm{i}k_n z}$, we consider the wave equation [15]

$$\nabla^2 \mathbf{E}_n(\mathbf{r}) + \frac{\omega_n^2}{c^2}\epsilon^{(1)}(\omega_n)\mathbf{E}_n(\mathbf{r}) = -\frac{\omega_n^2}{\epsilon_0 c^2}\mathbf{P}_n(\mathbf{r}).$$

For SHG, we define the polarization of the output field as $P_2(z) = \epsilon_0 \chi^{(2)} A_1^2 \mathrm{e}^{\mathrm{i}(k_1+k_2)z}$ and the wave equation becomes

$$\left[\frac{d^2 A_2}{dz^2} + 2\mathrm{i}k_2 \frac{dA_2}{dz} - k_2^2 A_2 + \frac{\omega_2^2}{c^2} n_2^2 A_2\right]\mathrm{e}^{\mathrm{i}k_2 z} = -\frac{\omega_2^2}{c^2}\chi^{(2)} A_1^2 \mathrm{e}^{2\mathrm{i}k_1 z}$$

with $k_2 = \omega_2 n_2/c$ and n_2 the index of refraction at ω_2. We then neglect the second derivative, which is small compared with the first one. This leads to

$$\frac{dA_2}{dz} = -\frac{\omega_2^2}{2\mathrm{i}k_2 c^2}\chi^{(2)} A_1^2 \mathrm{e}^{\mathrm{i}(2k_1-k_2)z}.$$

Then, the amplitude of the output field at the end of the effective thickness of the material L reads

$$A_2(L) = \frac{\mathrm{i}\omega_2^2 \chi^{(2)} c A_1^2}{2\omega_2 n_2 c^2}\int_0^L \mathrm{e}^{\mathrm{i}(2k_1-k_2)z} dz = \frac{\mathrm{i}\omega_2 \chi^{(2)} A_1^2}{2n_2 c}\left[\frac{\mathrm{e}^{\mathrm{i}(2k_1-k_2)L} - 1}{\mathrm{i}(2k_1 - k_2)}\right].$$

The corresponding field intensity is defined as $I_{i\omega} = 2n_i\epsilon_0 c|A_i|^2$. By ignoring the phase mismatch, the relation between the initial and final intensities is found to be

$$I_{2\omega} = 2n_2\epsilon_0 c\frac{\omega_2^2\left(\chi^{(2)}\right)^2 A_1^2 A_1^2}{4n_2^2 c^2}L^2 = \frac{\epsilon_0(2\omega_1)^2\left(\chi^{(2)}\right)^2 I_1^2 L^2}{2n_2 c 2n_1\epsilon_0 c 2n_1\epsilon_0 c} = \frac{\omega_1^2\left(\chi^{(2)}\right)^2 L^2}{2n_2 n_1^2 c^3 \epsilon_0} I_1^2.$$

The values of the refractive index are obtained from the theoretically calculated dielectric function. Using an analogous procedure, we can generalize this expression to

$$I_{s\omega} = \frac{s^2\omega_1^2\left(\chi^{(s)}\right)^2 L^2}{2^{s+1} n_{s\omega} n_\omega^s c^{s+1}\epsilon_0^{s-1}} I_\omega^s,$$

which relates the input and output intensities for s^{th} harmonic generation.

5. Carrier dynamics

Rather than studying a detailed state-by-state population dynamics, we consider an electron-energy-dependent average, in which we take into account the density of states $\rho(\varepsilon)$ (normalized per unit area and electron energy ε, see Sec. 6 below) as well as the population density $\Omega(\varepsilon)$ (defined such that $\Omega(\varepsilon) = 0$ at a fully unoccupied energy and $\Omega(\varepsilon) = \rho(\varepsilon)$ for full occupation). The latter satisfies the rate equation given in the main text, which also

incorporates the excitation rate $\Gamma_{ex}(\varepsilon, t)$ due to the pump laser pulse (see below) and the transition decay rates $\gamma(\varepsilon, \varepsilon')$ between different energy levels (with $\varepsilon > \varepsilon'$). The latter drive the subsequent dynamics. These rates involve in turn energy-dependent squared transition dipole matrix elements $d^2_{\varepsilon,\varepsilon'}$ averaged over the bands in the BZ (see Sec. 6 below). More precisely, $\gamma(\varepsilon, \varepsilon')$ is taken to be the sum of radiative and nonradiative components, where the former is directly proportional to $d^2_{\varepsilon,\varepsilon'}$ and mainly affects interband recombination processes, whereas the latter (taken as 1/(100 fs) if $0 < \varepsilon - \varepsilon' < 0.2$ eV) describes fast intraband relaxation.

To calculate the pumping excitation rate, we consider transitions between initial and final one-electron valence/conduction states denoted as $|m\mathbf{k}\rangle$ and $|n\mathbf{k}\rangle$, respectively, in which the Bloch momentum $\mathbf{k}$ is conserved as an approximation consistent with the fact that the momenta carried by the photons associated with radiative transitions are negligible. Although nonradiative processes are also argued to play a leading role in electron-hole recombination (see below), we maintain this simplification as a way to capture the main ingredients of carrier dynamics in a computational viable approach. The one-electron states are orthogonal solutions (i.e., $\langle \boldsymbol{nk}|\boldsymbol{mk}\rangle = \delta_{nm}$) of the noninteracting Hamiltonian $\widehat{\mathcal{H}}_0$, with associated energies defined by $\widehat{\mathcal{H}}_0|m\mathbf{k}\rangle = \hbar\varepsilon_{m\mathbf{k}}|m\mathbf{k}\rangle$ and $\widehat{\mathcal{H}}_0|n\mathbf{k}\rangle = \hbar\varepsilon_{n\mathbf{k}}|n\mathbf{k}\rangle$. The one-electron wave function $|n\mathbf{k}\rangle$ is perturbed by interaction with light (frequency ω, vector potential $\mathbf{A}$) as described by the Hamiltonian $\widehat{\mathcal{H}}_1 = (-i\hbar e/m^* c)\mathbf{A}.\nabla$, such that it is transformed into

$$|\Psi\rangle \approx |m\mathbf{k}\rangle e^{-i\varepsilon_{mk}t/\hbar} + \sum_n e^{-i\varepsilon_{nk}t/\hbar} C_{nm\mathbf{k}}|n\mathbf{k}\rangle.$$

We adopt the Coulomb gauge $\nabla.\mathbf{A} = 0$ combined with a vanishing scalar potential $\phi = 0$ in the absence of external charges. The time-dependent vector potential and electric field associated with a laser pulse are given by

$$\mathbf{A}(t) = \mathbf{A}_0 e^{-t^2/\Delta^2}\left(e^{-i\omega t} + \mathrm{c.c.}\right),$$

$$\mathbf{E}(t) = \mathbf{E}_0 e^{-t^2/\Delta^2}\left(e^{-i\omega t} + \mathrm{c.c.}\right),$$

where the electric field amplitude $\mathbf{E}_0 = (i\omega/c)\mathbf{A}_0$ is taken to be real. Solving the time-dependent Schrödinger equation $(\widehat{\mathcal{H}}_0 + \widehat{\mathcal{H}}_1)|\Psi\rangle = i\hbar\partial_t|\Psi\rangle$ in the $C_{nm\mathbf{k}} \ll 1$ limit (i.e., we are in the perturbative regime), we have to first order

$$i\hbar\dot{C}_{nm\mathbf{k}} = \left\langle n\mathbf{k}\middle|\widehat{\mathcal{H}}_1\middle|m\mathbf{k}\right\rangle e^{i(\varepsilon_{nk}-\varepsilon_{mk})t/\hbar}$$

for the wave function coefficients. Inserting the above expression for $\widehat{\mathcal{H}}_1$, we obtain

$$i\hbar\dot{C}_{nm\mathbf{k}} = -\frac{\hbar e}{m^*\omega}\mathbf{E}_0 \cdot \langle n\mathbf{k}|\nabla|m\mathbf{k}\rangle\, e^{i(\varepsilon_{n\mathbf{k}}-\varepsilon_{m\mathbf{k}})t/\hbar} e^{-t^2/\Delta^2}\left(e^{-i\omega t} + \mathrm{c.c.}\right),$$

where $\langle n\mathbf{k}|\nabla|m\mathbf{k}\rangle = -(m^*/\hbar)(\varepsilon_{n\mathbf{k}} - \varepsilon_{m\mathbf{k}})\langle n\mathbf{k}|\mathbf{r}|m\mathbf{k}\rangle$ and $\langle n\mathbf{k}|\mathbf{r}|m\mathbf{k}\rangle = \mathbf{d}_{nm\mathbf{k}}$ is the dipole matrix element associated with the electronic transition from initial to final states. In the rotating-wave approximation (i.e., neglecting off-resonance terms), and noticing that only terms $\varepsilon_{\mathrm{n}\mathbf{k}} - \varepsilon_{\mathrm{m}\mathbf{k}} \approx \omega$ are contributing, we can write

$$i\hbar\dot{C}_{nm\mathbf{k}} \approx e\,\mathbf{E}_0 \cdot \mathbf{d}_{nm\mathbf{k}} e^{i(\varepsilon_{n\mathbf{k}}-\varepsilon_{m\mathbf{k}}-\hbar\omega)t/\hbar} e^{-t^2/\Delta^2}.$$

Starting from $C_{nm\boldsymbol{k}}(-\infty) = 0$ in the infinite past and taking the integral of both sides of this equation, we find

$$C_{nm\boldsymbol{k}}(\infty) = -\frac{ie\sqrt{\pi}\Delta}{\hbar}\,\mathbf{E}_0 \cdot \mathbf{d}_{nm\mathbf{k}}\, e^{-(\varepsilon_{n\mathbf{k}}-\varepsilon_{m\mathbf{k}}-\hbar\omega)^2\Delta^2/4\hbar^2}.$$

Now, taking $\mathbf{E}_0$ along an in-plane direction $\hat{\mathbf{x}}$, the probability of finding the electron in the excited state $|n\mathbf{k}\rangle$ when starting from $|m\mathbf{k}\rangle$ reduces to

$$|C_{nm\boldsymbol{k}}(\infty)|^2 = \frac{\pi e^2\Delta^2}{\hbar^2} E_0^2\, |\hat{\mathbf{x}} \cdot \mathbf{d}_{nm\mathbf{k}}|^2\, e^{-(\varepsilon_{n\mathbf{k}}-\varepsilon_{m\mathbf{k}}-\hbar\omega)^2\Delta^2/2\hbar^2}.$$

Finally, we average $|\hat{\mathbf{x}} \cdot \mathbf{d}_{nm\mathbf{k}}|^2$ over the bands in the BZ to yield $d^2_{\varepsilon,\varepsilon'}$. where ε and ε' are the final and initial energies, respectively (see Sec. 6 below). Performing such an average in the above expression for the transition rate, denoting the initial and final energies as $\varepsilon' = \varepsilon_{m\mathbf{k}}$ and $\varepsilon = \varepsilon_{n\mathbf{k}}$, and integrating over ε' under the assumption that both $\rho(\varepsilon')$ and $d^2_{\varepsilon,\varepsilon'}$ have a smooth dependence on ε' (i.e., we just integrate the exponential $e^{-(\varepsilon-\varepsilon'-\hbar\omega)^2\Delta^2/2\hbar^2}$ and set $\varepsilon' = \varepsilon - \hbar\omega$ elsewhere), we find

$$\Gamma_{ex}(\varepsilon, t) = \frac{2\pi e^2 E_0^2}{\hbar}\, d^2_{\varepsilon,\varepsilon-\hbar\omega}\;\; e^{-2t^2/\Delta^2}$$

for the excitation rate to states of energy ε, in which we have included a normalized time-dependent Gaussian factor $(1/\Delta)\sqrt{2/\pi}\, e^{-2t^2/\Delta^2}$ incorporating the pump pulse profile. In the rate equation given in the main text, this expression is in turn multiplied by the density of occupied states $\Omega(\varepsilon - \hbar\omega)$ at the initial energy $\varepsilon - \hbar\omega$ times the density of available states $\rho(\varepsilon) - \Omega(\varepsilon)$ at the final energy ε.

6. Calculation of the density of states and dipole matrix elements

Using the DFT calculated energies $\varepsilon_{n\mathbf{k}}$, the density of states integrated over the unit cell of monolayer MoS_2 can be extracted as

$$\rho(\varepsilon) = V_{\mathrm{uc}} \sum_n \int_{\mathrm{BZ}} \frac{d\mathbf{k}}{(2\pi)^3} \delta(\varepsilon - \varepsilon_{n\mathbf{k}}) \approx \frac{1}{N_{\mathbf{k}} \sqrt{\pi}\, \sigma} \sum_{n,\mathbf{k}} e^{-(\varepsilon - \varepsilon_{n\mathbf{k}})^2/\sigma^2},$$

where n runs over all electron bands and V_{uc} is the unit cell volume. The rightmost expression is obtained by approximating the integral as a sum over a finite number $N_{\mathbf{k}}$ of $\mathbf{k}$ points in the BZ, with the delta function replaced by a narrow normalized Gaussian of width $\sigma = 50$ meV. The resulting density of states of monolayer MoS_2 is plotted in Fig. S4(a).

We also need the square of the dipole matrix elements as a function of initial and final energy states (ε_i and ε_f, respectively) averaged over the BZ. This quantity is given by (for polarization along x)

$$d^2_{\varepsilon,\varepsilon'} = |\langle \varepsilon | x | \varepsilon' \rangle|^2 = \frac{\sum_{n,m} \int_{\mathrm{BZ}} \delta(\varepsilon' - \varepsilon_{m\mathbf{k}}) \delta(\varepsilon - \varepsilon_{n\mathbf{k}}) |\langle n\mathbf{k} | x | m\mathbf{k} \rangle|^2}{\sum_{n,m} \int_{\mathrm{BZ}} \delta(\varepsilon' - \varepsilon_{m\mathbf{k}}) \delta(\varepsilon - \varepsilon_{n\mathbf{k}})},$$

which is computed by again substituting the delta functions by Gaussians. The transition dipole matrix elements $\langle n\mathbf{k} | x | m\mathbf{k} \rangle$ are obtained from DFT calculations. We plot the result for $d^2_{\varepsilon,\varepsilon'}$ as a function of initial and final energies in Fig. S4(b).

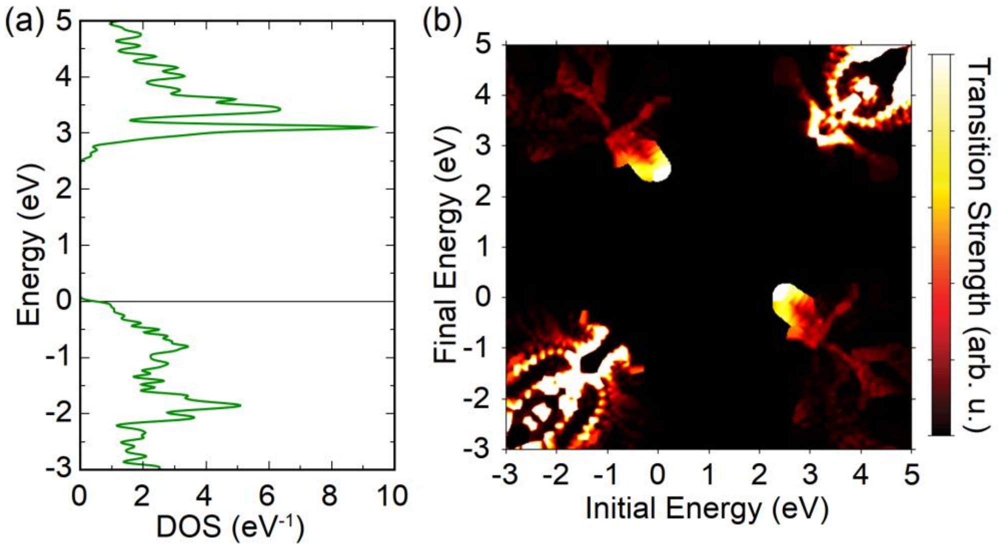

Figure S4. a) Density of states (ρ) of monolayer MoS_2 calculated for the unit cell. (b) Dipole matrix elements of monolayer MoS_2 averaged over the first Brillouin zone as a function of initial and final state energies.

7. Enhancement of recombination transition rates by coupling to excitons

To support the large correction factor for the recombination rates in the main text, we have calculated the enhancement in the local density of optical states (LDOS) at the film surface relative to its value in vacuum LDOS/LDOS$_0$, as this quantity is proportional to the decay rate of a dipolar emitter (in this case, involving electron-electron transitions combined with other excitations in the material). Evaluating the normalized LDOS by using the nonlocal parallel-momentum- and frequency-dependent surface conductivity $\sigma(k_\parallel,\omega)$ through the expression LDOS/LDOS$_0 \approx (3c^3/4\omega^3)\int_0^\infty k_\parallel^2 dk_\parallel \, \mathrm{Im}\{-(1+2\pi i k_\parallel \sigma(k_\parallel,\omega)/\omega)^{-1}\}$, where $\sigma(k_\parallel,\omega)$ is in turn calculated from first principles, we find LDOS/LDOS$_0$ ~ 8×10^5, which is ~250 times larger than the actual correction factor needed to phenomenologically account for the experimentally measured temporal evolution. A more realistic model should consider the effects of screening inside the material in which the excitation is taking place as well as the extension of the latter across the finite thickness of the layer ~0.65 nm. As an approximate approach to include such effects, we calculate the LDOS averaged over positions across the film; after some tedious but straightforward analysis, we find LDOS/LDOS$_0$ ~ 5×10^4, or equivalently, a factor of ~17 larger than the phenomenological value. This supports the need for a correction factor, although the LDOS overestimates it, possibly because it relates to point-dipole transitions rather than the finite-size states of the actual sample. We remark that the LDOS is dominated by the excitonic response, which enters this calculation through the nonlocal surface conductivity $\sigma(k_\parallel,\omega)$.

8. Optically controlled HHG in few-layer graphene

To demonstrate optically-controlled HHG in graphene, we fabricate a few-layer graphene sample following the mechanical exfoliation method, which is further characterized by Raman spectroscopy and atomic force microscopy (see Fig. S5). Figure S6(a) shows the 3rd- and 5th-

harmonic signals in graphene when the seed photon energy ($\hbar\omega_0$) is ≈ 0.56 eV with an average power of ≈ 60 μW (peak intensity of 846 GW/cm^2). The 3rd-HG signal at ~1.68 eV is much stronger than that of 5th-HG at ~2.81 eV. As shown in Fig. S6(b), the 5th-HG signal in the presence of control light ($\hbar\omega_c$ ≈ 3.1 eV, average power of 1 μW, corresponding to peak intensity of 14.1 GW/cm^2) first sharply decreases to reach a lowest modulation depth of 35% when Δτ ≈0.25 ps, and then starts recovering with a nonlinear trace that can be well fitted by a biexponential function with time constants of τ_1 ~0.15 ps and τ_2 ~2.0 ps. The fitting parameters match well with the ultrafast response of photon excited carriers in previously reported linear and nonlinear results in graphene [16-18].

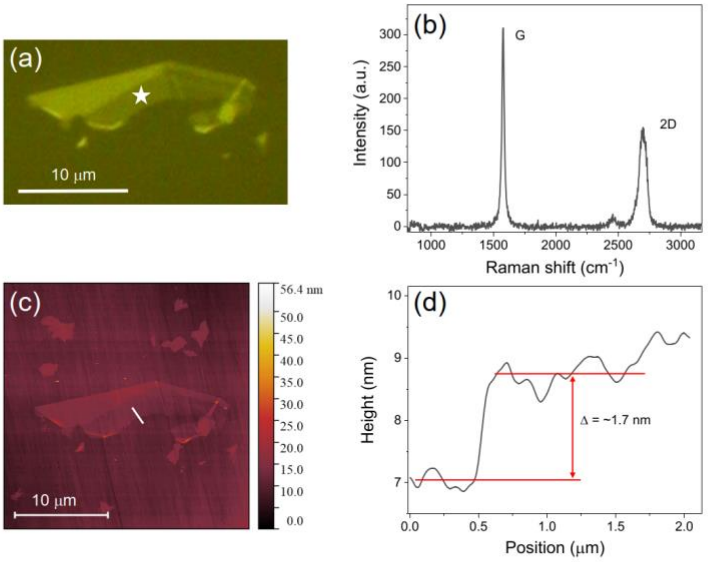

Figure S5. (a) Optical image of exfoliated few-layer graphene. (b) Raman spectrum at the position of the graphene flake marked by a star in (a). (c) Atomic force microscopy image shown a graphene flake. (d) Height profile with ~1.7 nm variation given by the white line in (c), indicating a ~5-layer graphene thickness.

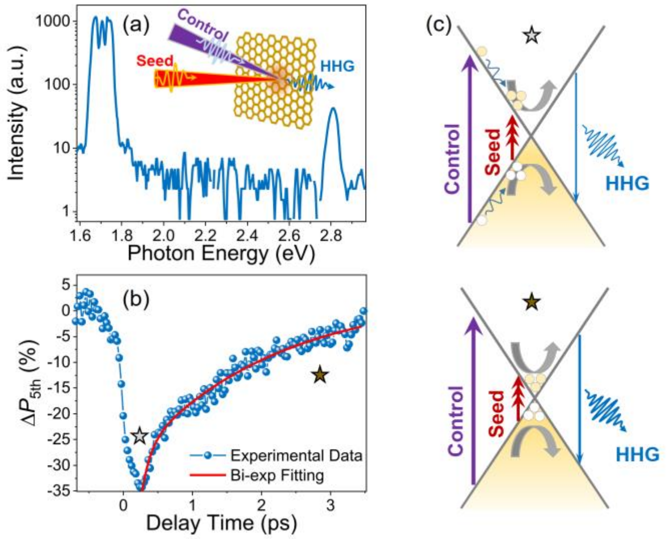

Figure S6. All-optical control of 5th-HG in graphene. (a) static (i.e., without control light) harmonics spectrum with the seed laser having $\hbar\omega_0 \approx 0.56$ eV and $P_0 = 60$ μW. Inset: simplified schematic of the optically controlled HHG process. (b) Time-resolved 5th-HG signal at $\hbar\omega_{5th} \approx 2.81$ eV in the presence of control light with power $P_c = 1$ μW. The stars mark different times coordinated with panel (c). (c) Simplified pictures of carrier dynamics when the delay time $\Delta\tau$ is ~0.2 ps (upper scheme) and of the order of a several ps (lower scheme).

Next, we describe the carrier dynamics in few-layer graphene in the light of the observed time-resolved HHG. Figure S6(c) illustrates the carrier dynamics induced by control light, which produces a modulation of the HHG signal. Before applying the control laser excitation, the HHG in pristine graphene is normally strong, mediated by excitation of Dirac fermions [19, 20]. With the control light, valence carriers are bleached by optical excitation to the conduction band, thus causing a reduction in the strength of HHG via the saturable absorption effect (upper scheme in Fig. S6(c)). The excited carriers in the high energy states then decay to lower-energy states through carrier-carrier relaxation within a fast recovery time τ_1 directly observed in HHG. Hot carriers finally relax via interband recombination, possibility mediated by phonons, with a slow decay time constant τ_2 (lower scheme in Fig. S6(c)) [21].

Reference

(1) P. Giannozzi *et al. J. Phys-Condens. Mat.* **2009,** 21 (39), 395502.
(2) D.R. Hamann. *Phys. Rev. B* **2013,** 88 (8), 085117.
(3) M. Schlipf, F. Gygi. *Comput. Phys. Commun.* **2015,** 196, 36-44.
(4) J. P. Perdew, K. Burke, M. Ernzerhof. *Phys. Rev. Lett.* **1996,** 77 (18), 3865-3868.
(5) Lars Hedin. *Phys. Rev.* **1965,** 139 (3A), A796.
(6) G. Onida, L. Reining, A. Rubio. *Rev. Mod. Phys.* **2002,** 74 (2), 601-659.

(7) D. Sangalli, A. Ferretti, H. Miranda, C. Attaccalite, I. Marri, E. Cannuccia, P. Melo, M. Marsili, F. Paleari, A. Marrazzo, G. Prandini, P. Bonfa, M. O. Atambo, F. Affinito, M. Palummo, A. Molina-Sanchez, C. Hogan, M. Gruning, D. Varsano, A. Marini. *J. Phys-Condens. Mat.* **2019,** 31 (32), 325902.
(8) A. Marini, C. Hogan, M. Gruning, D. Varsano. *Comput. Phys. Commun.* **2009,** 180 (8), 1392-1403.
(9) M. Rohlfing, S. G. Louie. *Phys. Rev. B* **2000,** 62 (8), 4927-4944.
(10) M. Palummo, O. Pulci, R. Del Sole, A. Marini, P. Hahn, W. G. Schmidt, F. Bechstedt. *J. Phys-Condens. Mat.* **2004,** 16 (39), S4313-S4322.
(11) Carlo A Rozzi, Daniele Varsano, Andrea Marini, Eberhard KU Gross, Angel Rubio. *Phys. Rev. B* **2006,** 73 (20), 205119.
(12) Raffaele Resta. *Rev. Mod. Phys.* **1994,** 66 (3), 899.
(13) Ivo Souza, Jorge Íniguez, David Vanderbilt. *Phys. Rev. B* **2004,** 69 (8), 085106.
(14) John Crank, Phyllis Nicolson Mathematical Proceedings of the Cambridge Philosophical Society, 1947; Cambridge University Press: pp 50-67.
(15) Robert W.Boyd, *Nonlinear Optics*. 2007.
(16) H. N. Wang, J. H. Strait, P. A. George, S. Shivaraman, V. B. Shields, M. Chandrashekhar, J. Hwang, F. Rana, M. G. Spencer, C. S. Ruiz-Vargas, J. Park. *Appl. Phys. Lett.* **2010,** 96 (8), 081917.
(17) W. Li, B. Chen, C. Meng, W. Fang, Y. Xiao, X. Li, Z. Hu, Y. Xu, L. Tong, H. Wang, W. Liu, J. Bao, Y. R. Shen. *Nano Lett.* **2014,** 14 (2), 955-959.
(18) Y. Cheng, H. Hong, H. Zhao, C. Wu, Y. Pan, C. Liu, Y. Zuo, Z. Zhang, J. Xie, J. Wang, D. Yu, Y. Ye, S. Meng, K. Liu. *Nano Lett.* **2020,** 20 (11), 8053-8058.
(19) N. Yoshikawa, T. Tamaya, K. Tanaka. *Science* **2017,** 356 (6339), 736-738.
(20) H. A. Hafez *et al. Nature* **2018,** 561 (7724), 507-511.
(21) M. Breusing, S. Kuehn, T. Winzer, E. Malić, F. Milde, N. Severin, J. P. Rabe, C. Ropers, A. Knorr, T. Elsaesser. *Phys. Rev. B* **2011,** 83 (15), 153410.
